# Expanding functional repertoires of fungal peroxisomes: contribution to growth and survival processes

**DOI:** 10.3389/fphys.2013.00177

**Published:** 2013-07-17

**Authors:** Jun-ichi Maruyama, Katsuhiko Kitamoto

**Affiliations:** Department of Biotechnology, The University of TokyoTokyo, Japan

**Keywords:** peroxisome, fungi, Woronin body, biotin, mitochondria

## Abstract

It has long been regarded that the primary function of fungal peroxisomes is limited to the β-oxidation of fatty acids, as mutants lacking peroxisomal function fail to grow in minimal medium containing fatty acids as the sole carbon source. However, studies in filamentous fungi have revealed that peroxisomes have diverse functional repertoires. This review describes the essential roles of peroxisomes in the growth and survival processes of filamentous fungi. One such survival mechanism involves the Woronin body, a Pezizomycotina-specific organelle that plugs the septal pore upon hyphal lysis to prevent excessive cytoplasmic loss. A number of reports have demonstrated that Woronin bodies are derived from peroxisomes. Specifically, the Woronin body protein Hex1 is targeted to peroxisomes by peroxisomal targeting sequence 1 (PTS1) and forms a self-assembled structure that buds from peroxisomes to form the Woronin body. Peroxisomal deficiency reduces the ability of filamentous fungi to prevent excessive cytoplasmic loss upon hyphal lysis, indicating that peroxisomes contribute to the survival of these multicellular organisms. Peroxisomes were also recently found to play a vital role in the biosynthesis of biotin, which is an essential cofactor for various carboxylation and decarboxylation reactions. In biotin-prototrophic fungi, peroxisome-deficient mutants exhibit growth defects when grown on glucose as a carbon source due to biotin auxotrophy. The biotin biosynthetic enzyme BioF (7-keto-8-aminopelargonic acid synthase) contains a PTS1 motif that is required for both peroxisomal targeting and biotin biosynthesis. In plants, the BioF protein contains a conserved PTS1 motif and is also localized in peroxisomes. These findings indicate that the involvement of peroxisomes in biotin biosynthesis is evolutionarily conserved between fungi and plants, and that peroxisomes play a key role in fungal growth.

## Introduction

Peroxisomes are ubiquitous organelles in eukaryotic cells and typically contain enzymes involved in the β-oxidation of fatty acids and detoxification of reactive oxygen species. Additionally, peroxisomes are known to have various physiological functions based on their roles in diverse metabolic activities. For example, mammalian peroxisomes participate in the lipid biosynthesis such as ether phospholipids, and in the oxidation of amino acids and polyamines (Wanders and Waterham, 2006). In plants, peroxisomes are involved in the glyoxylate cycle (Mano and Nishimura, 2005), photorespiration (Reumann and Weber, 2006), male-female gametophyte recognition (Boisson-Dernier et al., 2008) and biosynthesis of the hormones jasmonic acid and auxin (Weber, 2002; Woodward and Bartel, 2005). Peroxisomes also play important roles in higher eukaryotes, with defects in peroxisome biogenesis resulting in severe human disease, such as Zellweger syndrome, neonatal adrenoleukodystrophy, and Refsums disease (Waterham and Ebberink, 2012). In plants, loss of peroxisomal function causes embryo lethality, suggesting that peroxisomes have an essential role in growth and development (Hu et al., 2002; Schumann et al., 2003; Sparkes et al., 2003; Tzafrir et al., 2004; Fan et al., 2005).

The primary role of fungal peroxisomes is the β-oxidation of fatty acids, as fungal mutants lacking peroxisomes fail to grow in minimal medium containing fatty acids as the sole carbon source (Erdmann et al., 1989; Hynes et al., 2008). Peroxisomes are also required for methanol metabolism in methylotrophic yeasts, including *Pichia pastoris* (van der Klei et al., 2006). In filamentous fungi, peroxisomes are also involved in secondary metabolism including the biosynthesis of penicillin, AK (*Alternaria kikuchiana*) toxin, and paxilline (Saikia and Scott, 2009; Imazaki et al., 2010; Bartoszewska et al., 2011), plant pathogenicity (Kimura et al., 2001; Asakura et al., 2006), and sexual development (Bonnet et al., 2006; Peraza-Reyes et al., 2008). While fungal peroxisomes are known to proliferate massively on oleate and acetate, inducing substrates for this organelle (van der Klei and Veenhuis, 2006), it is apparent that many peroxisomes constitutively exist in the cell of filamentous fungi under the normal growth condition e.g., on glucose (Tanabe et al., 2011). The delayed growth and aberrant organelle morphologies observed in peroxisome-deficient mutants (Bonnet et al., 2006; Idnurm et al., 2007; Hynes et al., 2008) suggest that peroxisomes have fundamental roles for the growth of filamentous fungi. However, the molecular mechanisms underlying these severe growth effects remain unknown. In this review, evidence for the roles of peroxisomes in fungal growth, particularly the involvement of the Woronin body, a peroxisome-derived organelle with wound-healing function, and the recently identified function of peroxisomes in vitamin biosynthesis are presented.

## The woronin body, an organelle specific to pezizomycotina species, differentiates from peroxisomes

Species of Pezizomycotina (filamentous ascomycetes) grow via elongation of the hyphal tip to form straight primary hyphae with branches. The hyphae are divided into distinct cells by the formation of septa, and thus filamentous fungi are characterized by multicellularity. The septum is proposed to have several possible functions, including increasing the mechanical integrity of hyphae and division of mycelium into sections that undergo distinct developmental processes. However, septa do not completely separate adjacent cells in the hyphae due to the presence of a septal pore, which allows the passage of cytoplasm and organelles between adjacent cells (Markham, 1994; Freitag et al., 2004; Lew, 2005; Tey et al., 2005; Ng et al., 2009). This intercellular communication resembles that found in higher eukaryotes, such as gap junctions in animal cells and plasmodesmata in plant cells, and suggests that filamentous fungi possess a cell-to-cell channel that modulates responses to environmental changes and development processes necessary for multicellularity.

**Figure 1:**
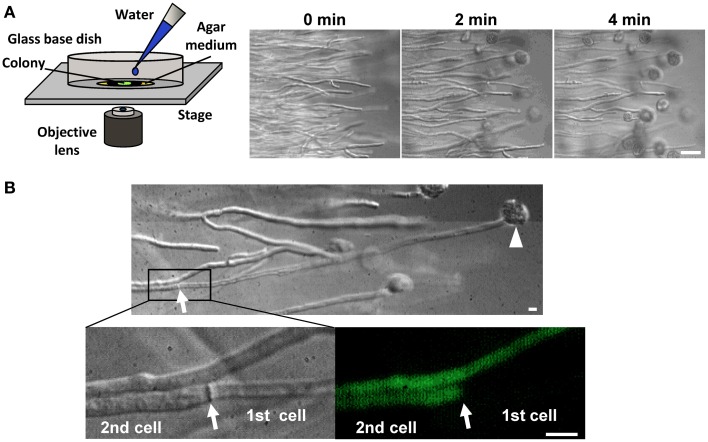
**Hyphal tip bursting upon hypotonic shock in the filamentous fungus *A. oryzae*. (A)** Time-lapse observation of hyphal tip bursting upon hypotonic shock. Hyphal tips at the edge of a colony grown on agar medium were observed by DIC microscopy before and after flooding hyphae with water. Bar: 50μm. **(B)** Excessive loss of cytoplasmic constituents is prevented upon hyphal tip bursting induced by hypotonic shock. The cytoplasm was labeled by EGFP. An arrowhead and arrow indicate a burst hyphal tip and the adjacent septum, respectively. Note that the cell (2nd) adjacent to the lysed cell (1st) retains its cytoplasmic constituents, as determined by DIC and fluorescence microcopy. Bar: 10μm.

Cytoplasmic continuity between adjacent cells through the septal pore is associated with catastrophic risk of cytoplasmic loss by adjacent cells due to hyphal lysis. This risk was clearly demonstrated by the exposure of the filamentous fungus *Aspergillus oryzae* grown on agar medium to hypotonic shock, which caused most of the hyphal tips to burst and lose the cytoplasmic constituents (Figure 1A) (Maruyama et al., 2005). However, as evidenced by differential interference contrast (DIC) and fluorescence microscopy, ~80% of the immediately adjacent cells retained their cytoplasmic constituents (Figure 1B) (Maruyama et al., 2005), allowing these cells to initiate regrowth by creating a new hyphal tip (Maruyama et al., 2006; Maruyama and Kitamoto, 2007). This process represents a type of defense system that aims to promote the survival of these multicellular organisms by preventing the excessive loss of cytoplasm upon hyphal lysis.

**Figure 2:**
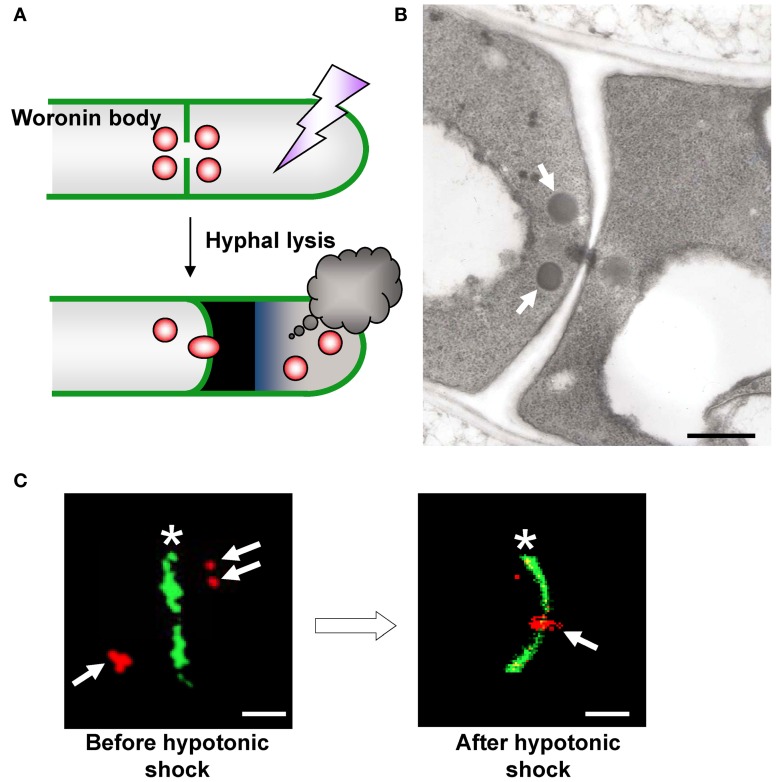
**Morphology and function of the Woronin body. (A)** Schematic model of Woronin body function. **(B)** Transmission electron microscopic observation of Woronin bodies (arrows) in *A. oryzae* (Maruyama et al., 2005). Bar: 500 nm. **(C)** Confocal images of Woronin bodies (red, arrows) and septa (green, asterisks) before (left) and after (right) hyphal tip bursting induced by hypotonic shock. Woronin bodies and septa were fluorescently labeled by expressing DsRed2–AoHex1 and RNase T1–EGFP fusion proteins, respectively (Maruyama et al., 2005). Bar: 2μm.

The Woronin body is a unique organelle present in Pezizomycotina species that plugs the septal pore upon hyphal lysis and prevents excessive cytoplasmic loss from the cell adjacent to the lysed cell (Figure 2A) (Markham and Collinge, 1987). This organelle has two morphologically distinct subclasses; it is generally observed by transmission electron microscopy as a spherical electron-dense structure in the vicinity of the septum (Figure 2B), although a limited number of species, such as *Neurospora crassa*, form hexagonal crystalline Woronin bodies that are occasionally visible by light microscopy (Markham, 1994).

Jedd and Chua (2000) first identified Hex1 as a major protein of the Woronin body in *N. crassa*. Genes encoding the Hex1 protein are conserved in Pezizomycotina species (Jedd and Chua, 2000; Asiegbu et al., 2004; Curach et al., 2004; Soundararajan et al., 2004; Maruyama et al., 2005; Beck and Ebel, 2013). Self-assembly of Hex1 provides the Woronin body with a mechanically solid core that provides resistance to the protoplasmic streaming pressure arising from hyphal lysis (Jedd and Chua, 2000; Yuan et al., 2003). Phosphorylation of Hex1 has a role in the formation of the multimeric core of the Woronin body (Tenney et al., 2000; Juvvadi et al., 2007). Deletion of the *hex1* gene results in the disappearance of Woronin bodies and is associated with severe cytoplasmic bleeding upon hyphal lysis (Jedd and Chua, 2000; Tenney et al., 2000; Maruyama et al., 2005). In the case of *A. oryzae*, *hex1* deletion (Δ*hex1*) significantly reduces the ability of this strain to prevent excessive cytoplasmic loss (Maruyama et al., 2005, 2010; Escaño et al., 2009). Using fluorescence microscopy, Woronin bodies were demonstrated to plug the septal pore adjacent to a lysed cell upon hyphal lysis in the *A. oryzae* wild-type strain (Figure 2C) (Maruyama et al., 2005). Recently, Bleichrodt et al. (2012) reported that the Woronin body reversibly closes the septal pore during normal growth of *A. oryzae*, a function that contrasts the behavior of this organelle conventionally observed during hyphal lysis. In addition, although wild-type *A. oryzae* has heterogeneous distribution of hyphae and gene expression activity, the absence of Woronin bodies results in uniform activity distribution of different cells (Bleichrodt et al., 2012). Collectively, Woronin bodies impede cytoplasmic continuity between adjacent cells during normal growth and help maintain hyphal heterogeneity in mycelia. This function of Woronin bodies may represent the most primitive way to regulate cell-to-cell channels in multicellularity by a simple plugging behavior similar to that upon hyphal lysis. Additionally, the roles of Woronin bodies in conidiation (asexual spore formation), survival under nitrogen starvation and efficient plant pathogenesis were reported (Yuan et al., 2003; Soundararajan et al., 2004).

**Figure 3:**
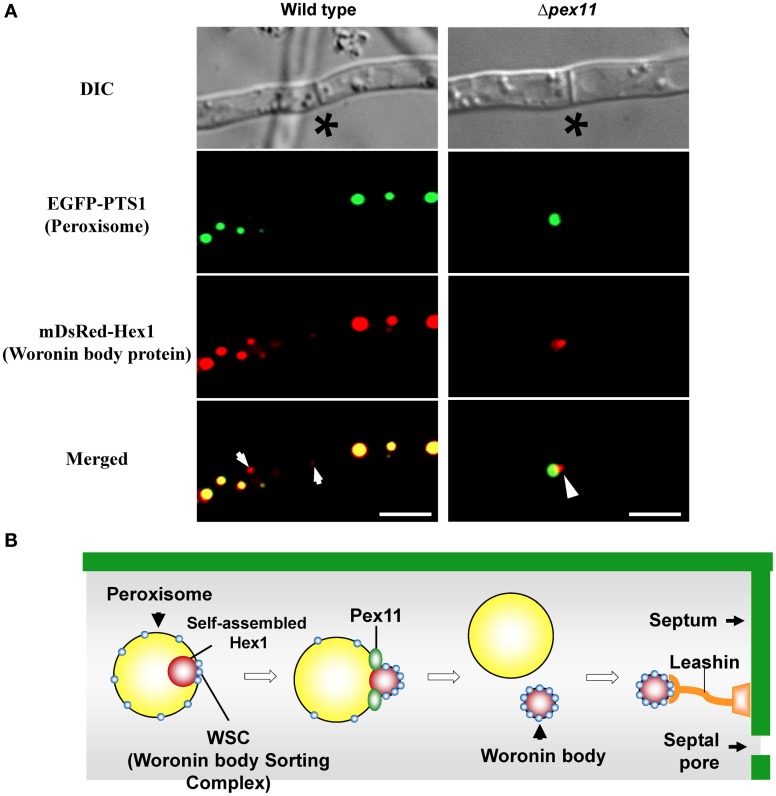
**Differentiation of the Woronin body from peroxisomes. (A)** Fluorescence microscopic analysis of wild-type and Δ*pex11* strains of *A. oryzae* expressing EGFP-PTS1 and mDsRed-AoHex1 fusion proteins for visualization of peroxisomes and the major Woronin body protein, respectively (Escaño et al., 2009). Asterisks denote septa and arrows indicate Woronin bodies (red) independent of peroxisomes (green). Arrowheads represent assembly of Hex1 attached to the matrix side of the peroxisome. Bars: 5 μm. **(B)** Schematic model of Woronin body differentiation from peroxisomes.

A relationship between peroxisomes and the Woronin body is suggested from the fact that Hex1 contains peroxisomal targeting signal sequence 1 (PTS1) at the C-terminus (Jedd and Chua, 2000). Time-lapse imaging demonstrated that Woronin bodies bud from peroxisomes in *N. crassa* (Tey et al., 2005) and that Woronin body biogenesis requires the presence of peroxins that mediate peroxisomal protein import (Ramos-Pamplona and Naqvi, 2006; Managadze et al., 2007; Liu et al., 2008). The peripheral membrane peroxisomal protein Pex11 is implicated in peroxisomal proliferation and division (Erdmann and Blobel, 1995; Marshall et al., 1995), and in the absence of Pex11, filamentous fungi only contain few enlarged peroxisomes (Figure 3A, EGFP-PTS1) (Hynes et al., 2008; Escaño et al., 2009; Opaliński et al., 2012). It was also demonstrated that ability of Pex11-deficient strain of *A. oryzae* to prevent the excessive loss of cytoplasm is reduced by ~30% compared to wild type (Escaño et al., 2009), indicating that Pex11 is involved in Woronin body function. Under fluorescence microscopy, Woronin bodies are typically observed as small dots independent of peroxisomes (Figure 3A, mDsRed-AoHex1). In the absence of Pex11, however, the Woronin body protein Hex1 forms a structure that attaches to the matrix side of the peroxisomal membrane, but the mature Woronin body fails to differentiate from peroxisomes (Figure 3A) (Escaño et al., 2009). The Pezizomycotina-specific protein WSC (Woronin body sorting complex) recruits the Hex1 assembly to the matrix side of the peroxisomal membrane and facilitates the budding of the Woronin body (Liu et al., 2008). It has been suggested that Pex11 elongates the peroxisomal membrane to facilitate the division of peroxisomes by dynamin-related proteins (Koch et al., 2003, 2004; Schrader, 2006). Heterologous expression of Hex1 in the yeast *Saccharomyces cerevisiae* suggested that dynamin-related proteins participate in the budding of Woronin bodies from peroxisomes (Würtz et al., 2008). ApsB, a component of the microtubule-organizing center (MTOC), has been shown to interact with Hex1 and to localize to peroxisomes via peroxisomal targeting signal sequence 2 (PTS2) (Zekert et al., 2010). Hex1 physically associates with the essential matrix import peroxin Pex26 and promotes the enrichment of Pex26 in the membranes of differentiated peroxisomes (Liu et al., 2011). After Woronin bodies differentiate from peroxisomes, evidence suggests that the Pezizomycotina-specific protein Leashin (LAH) tethers the Woronin bodies to the vicinity of the septum (Ng et al., 2009). A schematic model of Woronin body differentiation from peroxisomes is presented in Figure 3B. Although a number of proteins functionally/spatially related to the Woronin body have been identified (Engh et al., 2007; Fleissner and Glass, 2007; Kim et al., 2009; Maruyama et al., 2010; Lai et al., 2012; Yu et al., 2012), the molecular mechanism for Woronin body biogenesis remains to be completely resolved.

## Involvement of peroxisomes in biotin biosynthesis in fungi

**Figure 4:**
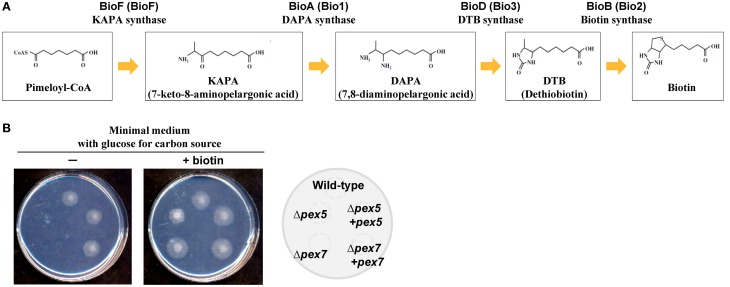
**Biotin biosynthetic pathway and biotin auxotrophy in peroxisome-deficient strains. (A)** The final four reactions of the biotin biosynthetic pathway and involved enzymes from fungi and plants (in parentheses). **(B)** Growth impairment of strains defective in peroxisomal targeting signal receptors (Δ*pex5* and Δ*pex7*) grown on minimal medium containing glucose as the sole carbon source. Growth of the wild-type, Δ*pex5*, and Δ*pex7* strains and the complemented strains (indicated to the right) on medium with and without biotin.

Biotin is an essential cofactor involved in a number of carboxylation and decarboxylation reactions (Knowles, 1989). In eukaryotes, plants and numerous fungal species are capable of synthesizing biotin. The studies of plants and fungi have revealed that the final four reactions in the biosynthetic process, which convert pimeloyl-CoA to biotin, are conserved (Figure 4A) (Streit and Entcheva, 2003). In plants, the enzymes BioF, Bio1, Bio3, and Bio2 mediate the final four steps of biotin biosynthesis. It was previously reported that BioF, a 7-keto-8-aminopelargonic acid (KAPA) synthase catalyzing the conversion of pimeloyl-CoA to KAPA, is localized to the cytoplasm (Pinon et al., 2005). The final three reactions converting KAPA to biotin occur in mitochondria. The *BIO3* and *BIO1* genes are unidirectionally aligned and expressed as a chimeric transcript, resulting in the production of Bio3-Bio1 as a bifunctional protein catalyzing desthiobiotin (DTB) synthase and 7, 8-diaminopelargonic acid (DAPA) synthase reactions (Muralla et al., 2008). Bio3-Bio1 contains a mitochondrial targeting sequence (MTS) and localizes in mitochondria (Muralla et al., 2008; Cobessi et al., 2012). The Bio2 protein, a biotin synthase catalyzing the conversion of DTB to biotin, also contains a MTS and must be mitochondrially localized for biotin prototrophy (Baldet et al., 1997; Picciocchi et al., 2001; Arnal et al., 2006). It was therefore suggested that plant biotin biosynthesis occurs in both the cytoplasm and mitochondria (Rébeillé et al., 2007).

**Figure 5:**
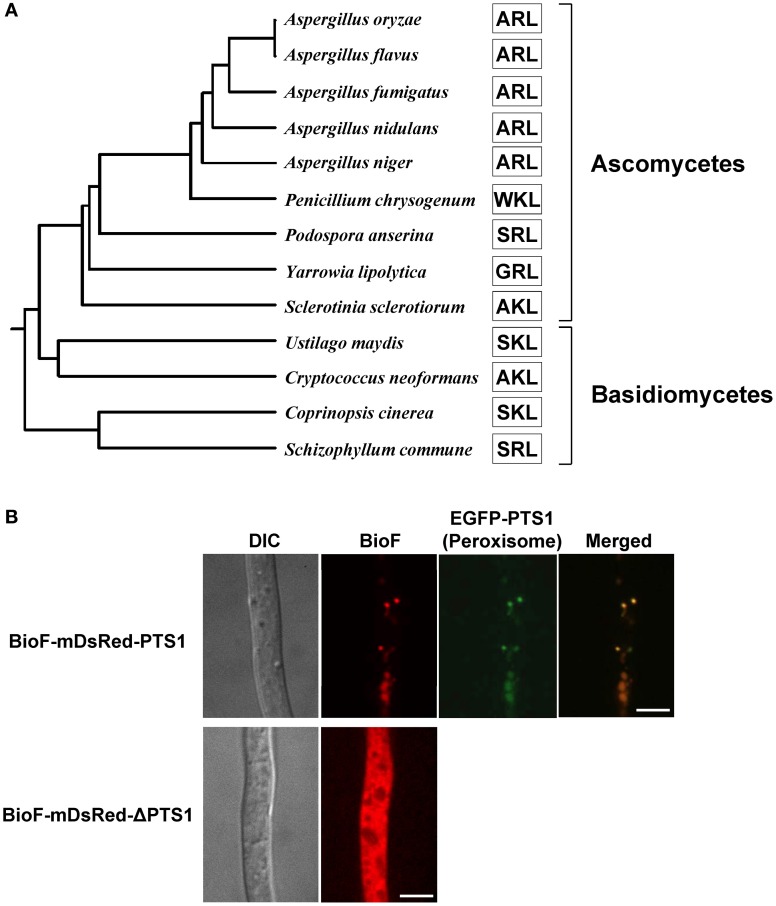
**Phylogenetic relationship and peroxisomal localization of fungal BioF proteins. (A)** Phylogenetic analysis of fungal BioF proteins. The amino acid residues of the C-terminal peroxisomal targeting signals (PTS1) are indicated by open boxes. The full-length amino acid sequences of the fungal BioF proteins were aligned using the Clustal W program (version 2.1), and then the phylogenetic tree was constructed. The Genbank accession numbers for the sequences used in the analysis are as follows: *Aspergillus oryzae*, XP_001817022.1; *Aspergillus flavus*, XP_002383037.1; *Aspergillus fumigatus*, XP_747713.1; *Aspergillus nidulans*, ACR44939.1; *Aspergillus niger*, XP_001396737.1; *Penicillium chrysogenum*, XP_002563821.1; *Podospora anserina*, XP_001903515.1; *Yarrowia lipolytica*, XP_504066.1; *Sclerotinia sclerotiorum*, XP_001590700.1; *Ustilago maydis*, XP_757344.1; *Cryptococcus neoformans*, XP_566616.1; *Coprinopsis cinerea*, XP_001836705.2; and *Schizophyllum commune*, XP_003028193.1. **(B)** Peroxisomal localization of fungal BioF protein. Note that BioF (BioF-mDsRed-PTS1) co-localizes with peroxisomes (EGFP-PTS1), but disperses in the absence of PTS1 (BioF-mDsRed-ΔPTS1) (Tanabe et al., 2011). Bars: 5 μm.

In *Aspergillus* species, mutants of the *pex5* and *pex7* genes are defective in protein import into the peroxisomal matrix due to the lack of PTS1 and PTS2 receptors, respectively (Hynes et al., 2008; Tanabe et al., 2011). These mutants fail to grow in minimal medium containing oleic acid as the sole carbon source due to defective peroxisomal β-oxidation; however, unlike the corresponding yeast mutants, the mutants of *Aspergillus* species also exhibit growth defects when grown on glucose (Hynes et al., 2008; Tanabe et al., 2011). Surprisingly, the growth defects are restored by the addition of biotin (Figure 4B). In fungi, biotin is synthesized through the sequential activities of three Bio proteins: BioF, a KAPA synthase; BioD/A, a chimeric protein composed of DTB and DAPA synthases; and BioB, a biotin synthase (Figure 4A) (Magliano et al., 2011a,b; Tanabe et al., 2011). The BioD/A protein localizes in mitochondria, suggesting that this is where KAPA is converted to biotin (Tanabe et al., 2011). Protein sequence analysis of fungal BioF proteins revealed that the C-terminal PTS1 sequences are conserved in ascomycete and basidiomycete species (Figure 5A). Consistent with these findings, BioF protein localizes in the peroxisomes via PTS1 (Figure 5B), and the peroxisomal targeting of this KAPA synthase is required for biotin biosynthesis (Magliano et al., 2011a; Tanabe et al., 2011). Yeast species appear to have lost the gene encoding BioF, as evidenced by their biotin auxotrophy, although several species have reacquired biotin prototrophy by horizontal gene transfer and gene duplication followed by neofunctionalization (Hall and Dietrich, 2007).

**Figure 6:**
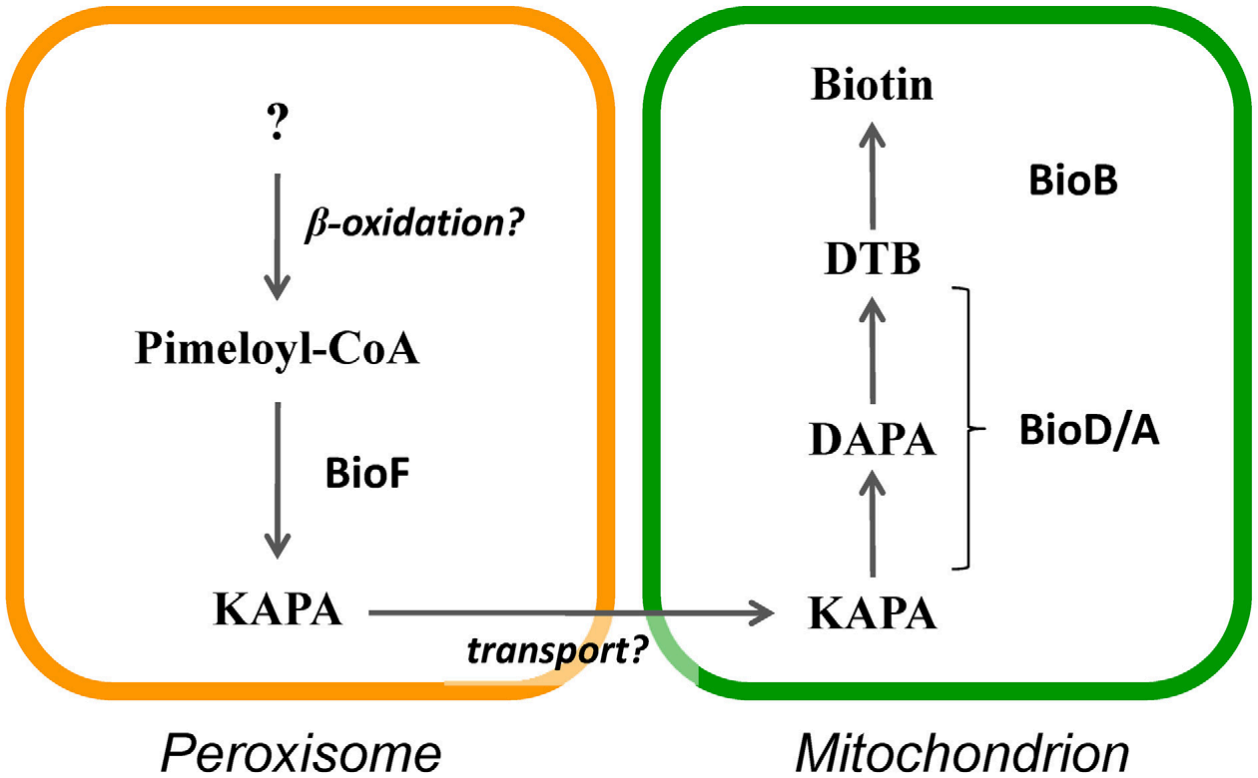
**Model of subcellular compartmentalization of the biotin biosynthetic pathway in eukaryotes**.

A new model for biotin biosynthesis in fungi is proposed in Figure 6. In this biotin biosynthesis pathway, the production of pimeloyl-CoA may involve proteins containing PTS1 and PTS2 (Tanabe et al., 2011), while peroxisomal β-oxidation is also involved (Magliano et al., 2011a). Ohsugi et al. (1988) reported that pimelic acid, a putative pimeloyl-CoA precursor, were produced from long chain fatty acids such as oleic acid in yeasts, which may support a relevance of β-oxidation to supplying a precursor substrate for biotin biosynthesis. In peroxisomes, KAPA is first synthesized from pimeloyl-CoA by BioF protein and is then likely transported from peroxisomes to mitochondria, where it serves as a substrate for the final series of biosynthesis reactions that convert KAPA to biotin by the action of the BioD/A and BioB proteins. Thus, functionally coupling between peroxisomes and mitochondria appears to be required for biotin biosynthesis.

## Conserved peroxisomal localization of biof protein and its possible relevance to fungal growth/developmental processes

**Figure 7:**
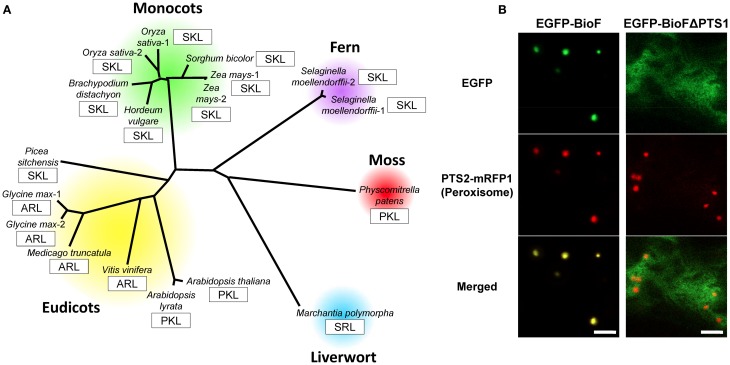
**Phylogenetic relationship and peroxisomal localization of plant BioF proteins. (A)** Phylogenetic analysis of plant BioF proteins (Maruyama et al., 2012). The amino acid residues of C-terminal peroxisomal targeting signals (PTS1) are indicated by open boxes. The full-length amino acid sequences of the plant BioF proteins were aligned using the method described in Figure 5. The Genbank accession numbers for the sequences used in the analysis are as follows: *Arabidopsis thaliana*, NP_974731.1; *Arabidopsis lyrata*, XP_002871105.1; *Oryza sativa*-1, BAD87813.1; *Oryza sativa*-2, NP_001065381.1; *Hordeum vulgare*, BAK03504.1; *Brachypodium distachyon*, XP_003574335.1; *Sorgham bicolor*, XP_002467492.1; *Zea mays*-1, ACG35792.1; *Zea mays*-2, ACG35881.1; *Selaginella moellendorffii*-1, XP_002969752.1; *Selaginella moellendorffii*-2, XP_002981364.1; *Physcomitrella patens*, XP_001769874.1; *Picea sitchensis*, ABR18106.1; *Vinis vinifera*, XP_002268950.1; *Medicago truncatula*, XP_003598166.1; *Glycine max*-1, XP_003527547.1; and *Glycine max*-2, XP_003522881.1. The amino acid sequence of the BioF protein of *Marchantia polymorpha* was confirmed by PCR amplification and cDNA sequencing based on information obtained from the *Marchantia* expression sequence tag database (Maruyama et al., 2012). **(B)** Peroxisomal localization of plant BioF protein. Note that BioF (EGFP-BioF-PTS1) co-localizes with the peroxisomes (PTS2-mRFP1), but disperses in the absence of PTS1 (EGFP-BioF-ΔPTS1) (Tanabe et al., 2011). Bars: 5 μm.

As described above, plant BioF protein functions as a KAPA synthase and was shown to be cytosolic by GFP fusion at the C-terminus (Pinon et al., 2005). Phylogenetic analysis revealed that BioF proteins from various plant species possess PTS1 at the C-terminus (Figure 7A) (Tanabe et al., 2011; Maruyama et al., 2012), suggesting that the peroxisomal localization of BioF proteins is conserved throughout the plant kingdom. An N-terminal GFP-BioF fusion protein co-localizes with peroxisomes, and deletion of PTS1 causes cytosolic localization, suggesting that BioF is localized to peroxisomes via the PTS1 sequence (Figure 7B) (Tanabe et al., 2011).

Plant biotin-auxotrophic mutants exhibit embryo lethality, indicating that biotin biosynthesis is vital for plant growth and development (Schneider et al., 1989; Shellhammer and Meinke, 1990; Patton et al., 1998; Tzafrir et al., 2004; Arnal et al., 2006). Embryo development also requires peroxisomal functions (Hu et al., 2002; Schumann et al., 2003; Sparkes et al., 2003; Tzafrir et al., 2004; Fan et al., 2005). Tanabe et al. (2011) suggested that fungi and plants use an evolutionarily conserved pathway for biotin biosynthesis that involves both peroxisomes and mitochondria. These findings suggest that biotin biosynthesis might be one of the reasons why peroxisomal deficiency results in embryo lethality. The *Aspergillus* peroxisome-deficient strains showing biotin auxotrophy exhibit abnormal polar growth (Tanabe et al., 2011), and impairment of sexual development by peroxisomal malfunction was reported in filamentous fungi (Bonnet et al., 2006). These similarities in the fungal and plant phenotypes indicate that growth and developmental defects due to peroxisomal deficiency may be partially or entirely attributed to biotin auxotrophy. More extensive studies will provide insight into the importance of biotin biosynthesis and peroxisomal function during growth and development of fungi and plants.

## Conclusion

The primary function of fungal peroxisomes was long thought to be limited to the β-oxidation of fatty acids. During the two past decades, an increasing number of studies have unmasked the functional diversity of fungal peroxisomes (Pieuchot and Jedd, 2012), including the very recent findings that peroxisomes contain siderophore biosynthetic enzymes and are involved in iron acquisition (Gründlinger et al., 2013), and that several glycolysis enzymes possess cryptic PTS1 motifs that are activated by alternative splicing and stop codon read-through (Freitag et al., 2012). The present review has described the findings that demonstrate the fundamental involvement of fungal peroxisomes in the regulation of multicellular growth and the biosynthesis of the essential vitamin biotin. Further investigations, including proteomic/metabolomic approaches and genomic bioinformatics, will lead to a comprehensive understanding of the newly emerged functions of fungal peroxisomes.

### Conflict of interest statement

The authors declare that the research was conducted in the absence of any commercial or financial relationships that could be construed as a potential conflict of interest.
